# Maternal and Neonatal Outcomes Associated With COVID-19 Infection in Pregnant Mothers Admitted in Tertiary Care Hospital in Central State of India

**DOI:** 10.7759/cureus.38235

**Published:** 2023-04-28

**Authors:** Neha Singh, Jyoti Jaiswal, Nikita Sherwani, Tripti Nagaria, Onkar Khandwal, Arvind Neral

**Affiliations:** 1 Department of Microbiology, Pt. Jawahar Lal Nehru Memorial Medical College, Raipur, IND; 2 Department of Obstetrics and Gynecology, Pt. Jawaharlal Nehru Memorial Medical College, Raipur, IND; 3 Department of Obstetrics and Gynecology, Pt. Jawahar Lal Nehru Memorial Medical College, Raipur, IND; 4 Department of Pediatrics, Pt. Jawahar Lal Nehru Memorial Medical College, Raipur, IND; 5 Department of Pathology, Pt. Jawahar Lal Nehru Memorial Medical College, Raipur, IND

**Keywords:** sars-cov-2, covid-19, coronavirus, neonatal outcome, vertical transmission, feto-maternal outcome, pregnancy outcomes, maternal outcomes

## Abstract

In spite of various reports on perinatal outcomes of coronavirus disease 2019 (COVID-19) during pregnancies, the effects of severe acute respiratory syndrome coronavirus 2 (SARS-CoV-2) on unborn babies and pregnant mothers are still mysterious. The goal of our research is to examine the perceived fetomaternal outcomes of COVID-19 during pregnancy. A total of 396 pregnant women were admitted to the Department of Gynaecology and Obstetrics, Pt. JNM Medical College, Raipur, Chhattisgarh, India, during the period from July 20, 2020 to January 6, 2021. The presence of SARS-CoV-2 in different biological samples was recorded via positive quantitative reverse transcriptase-polymerase chain reaction (RT-PCR) test results. All the newborns delivered from the infected pregnant mothers were tested as RT-PCR negative. Negative findings of RT-PCR for respiratory swabs of newborns, amniotic fluid, placental tissue, breast milk, vaginal swabs, and cord blood indicated no transmission of the virus from mother to baby. However, maternal outcomes, such as hospitalization (46.96%), preeclampsia (13.88%), pre-term birth (14.39%), prelabor rupture of membranes (PROM) before 34 weeks (3.78%), PROM before 37 weeks (2.77%), vaginal bleeding (4.29%), postpartum hemorrhage (2.52%), pregnancy-induced hypertension (1.51%), and neonatal outcomes such as low birth weight ≤1.5 kg (6.59) and 1.6-2.4 kg (39.34%), intrauterine deaths (IUD) (0.50%), fetal distress (22.33%), NICU admission (5.58%), meconium-stained liquor (14.46%), diarrhea (0.25%), and low APGAR score 4-6 at 1 min (20.54%), were observed. The results of the present study indicate that SARS-CoV-2-induced complications during pregnancy must be taken seriously. Intrauterine fetal deaths occurred at lower rates. There is no substantial proof of vertical perinatal transmission of the virus, as none of the neonates had tested positive for COVID-19.

## Introduction

In 2020, the number of severe acute respiratory syndrome coronavirus 2 (SARS-CoV-2) cases worldwide increased significantly [1]. By October 14, 2022, there have been 620,878,405 confirmed cases of COVID-19, including 6,543,138 deaths, reported to the WHO; more than 528,905 fatalities (COVID-19) have occurred only in India alone [2]. It is now obvious that this virus is particularly dangerous and provides a particular risk to those who are already at risk, such as the elderly, immunosuppressed, and individuals with specific pre-existing diseases; in addition, pregnant women may also be more vulnerable, especially those in the third trimester [3]. Due to immunological and physical changes, such as altered T-cell immunity, a reduction in lung capacity, and a reduction in functional residual capacity, pregnant women are generally more susceptible to almost all respiratory infections [4]. Due to concerns about how COVID-19 can affect unborn or newborn children and pregnant women who were seen as being in a high-risk group various short- and long-term studies being conducted all around the world [5]. A progressively clearer picture of how COVID-19 affects pregnancy outcomes has emerged as fresh research has accumulated rapidly. Some research findings showed that SARS-CoV-2 may affect pregnant women at any stage of the pregnancy and may potentially have adverse effects on both the mother and the newborns. There is a lack of information regarding pregnancy outcomes in women infected during the later stages of pregnancy, despite the fact that few studies included women infected during various stages of pregnancy. Pregnancy outcomes for the SARS-CoV-2 infection were reported for the group as a whole and not by trimester of infection. Preterm birth and caesarian deliveries were more common in women who contracted the flu in the latter stages of pregnancy, according to earlier investigations conducted on influenza infection during pregnancy. Other coronaviruses, such as the Middle East Respiratory Syndrome Coronavirus (MERS-CoV) and the Severe Acute Respiratory Syndrome Coronavirus (SARS-CoV), have been linked to an increased risk of spontaneous miscarriage and preterm birth. Scientists have tried to explore the occurrence of SARS-CoV-2 infection during the third trimester of pregnancy and its adverse outcomes, evaluating loads of the virus in respiratory, blood, breastmilk, and placental tissue samples, as well as investigating the development of maternal antibodies and, how well those antibodies passed through the placenta to the fetus (an indicator of potential immune protection from the mother); however, these results are limited and still being composed and evaluated for establishing a solid conclusion [6]. In line with an earlier report, the scientists have also established that while the placenta expresses major molecules ACE-2 receptor and the TMPRSS2 enzyme which is used by SARS-CoV-2 to cause infection in the placental tissues. These two molecules are rarely expressed together in the same location [7]. Due to the presence of ACE-2 molecules on the placental surface, it is more prone to get viral infection and also responsible for placental inflammation and adverse outcomes during pregnancy. In the current investigation, a population-based analysis was conducted to determine whether SARS-CoV-2 infection during pregnancy could have an impact on the course of the pregnancy or lead to utero vertical transmission; this data would provide Important information for the safety of women and children. The link between SARS-CoV-2 infection during later pregnancies and the risk of negative outcomes, such as preterm birth, low birth weight, PROM, newborn hypoxia, and cesarean section, was investigated for the first time in our cohort study in Chhattisgarh, a central state of India. Moreover, it was investigated if any possible in-utero vertical transmission of COVID-19 occurred.

## Materials and methods

A total of 396 pregnant women were admitted to the Department of Gynaecology and Obstetrics, Pt. JNM Medical College, Raipur, Chhattisgarh, India. Recruitments were carried out during the period from July 20, 2020 to January 6, 2021 and at the time of delivery. Oropharyngeal and nasopharyngeal swabs, amniotic fluid, placental tissue, breast milk, vaginal swabs, and cord blood samples were collected from the delivering mothers and submitted to the state key virology and research diagnostic laboratory to diagnose COVID-19 infection. All the pregnant women tested positive for SARS-CoV-2 infection, which was confirmed via a real-time reverse-transcriptase polymerase chain reaction (RT-PCR) assay [8,9]. All the pregnant women were tested due to either occurrence of symptoms or exposure, as they were residing or living in the hotspot/cluster/containment zone/among evacuee areas. Demographic profiles and other clinical histories were recorded for all admitted patients. Nasopharyngeal and oropharyngeal swabs were collected from the expectant mothers at the time of delivery for RT-PCR, whereas nasopharyngeal and oropharyngeal swabs from newborns were collected within 0-12h of delivery. Specimens were collected in viral transport medium (Biopro, Alchem Diagnostic, Coimbatore, India) and validated and approved by ICMR, NIV, Pune, India, and VTM kits equipped with universal viral transport sterile swab applicators. All pregnant mothers and health workers wore full-protective gear (PPE) inside the labor room to avoid airborne or any other environmentally generated transmission of viral infection. Viral nucleic acids from all samples were extracted (RNA Extraction kit COVID-19, HUWELL, Hyderabad, India) via manual extraction, followed by RT-PCR testing by using a readymade kit (Quantiplus Multiplex COVID-19 Detection, V2.0, HUWELL, India) approved by ICMR, India. Test results were confirmed by pre-designed primers for the E gene and N genes. The primer sequence for the N gene was GTTTGGTGGACCCTCAGATTGGTGAACCAAGACGCAGTATHEXTGGGTAAACCTTGGGGCCGACGTTGT-BHQ (WT) and the primer for the E gene was CENR: ATCGAAGCGCAGTAAGGATGCENP: FAMTGCTTTCGTGGTATTCTTGCTAGTTACACTBHQCENF: CGGAAGAGACAGGTACGTTAATAG. Maternal outcomes, such as asymptomatic and symptomatic responses, mode of delivery, preterm, PROM, morphological changes in the placenta, postpartum hemorrhage, hospitalization, admission to ICU, maternal death, vaginal bleeding, the need for oxygen, preeclampsia, thromboembolic events, and pregnancy-induced hypertension, were recorded. In addition, Neonatal outcomes, such as weight of the fetus, fetal distress, NICU admission, congenital anomaly, meconium-stained liquor, post-natal infection, fever, diarrhea, lethargy, IUD, and APGAR score, were also recorded. Direct breastfeeding was proactively encouraged in these patients while taking all appropriate safety precautions, including acquiring an N95 mask, and gloves, cleaning one's hands, and using hand sanitizer [10]. All results were entered into a Microsoft Excel Sheet; the continuous variables were expressed as standard deviation (SD) and mean, while all categorical variables were expressed as frequency and percentages.

## Results

Demographic data and comorbidities

Table 1 showed the RT-PCR findings of various biological samples collected at the time of delivery. All nasopharyngeal and oropharyngeal swab samples collected from the 396 pregnant mothers were found to be positive for SARS-CoV-2 against selected genes, whereas other biological samples, such as amniotic fluid, placental tissue, breast milk, vaginal swabs, and cord blood, came back negative.

**Table 1:** RT-PCR findings of in various biological samples collected at the time of delivery.

Samples	Positive findings	Negative findings
Pregnant women (n=396)		
Naso&oropharyngeal swabs	+ve	
Amniotic fluid		-ve
Placental tissue		-ve
Breast milk		-ve
Vaginal swabs		-ve
Cord blood		-ve
Newborn (n=394)		
Naso & oropharyngeal swabs		-ve

All of the babies born to positive mothers tested negative. Table 2 shows the demographic profile of the admitted pregnant women. The majority of enrolled women were belonging to the age group of 20-25 years (n=169; 42.67%), and 26-30 years (n= 122; 30.80%). The highest education level of the registered women was with higher secondary (n=146; 36.89%) and most of them were housewives (n=390; 98.48%). The gestational age of the admitted mothers at the time of delivery was recorded as 34-37 weeks (n=159;40.15 %), 28-34 weeks (n=122; 30.80%), >37 weeks (n=101; 25.50%), and <28 weeks (n=14; 3.53%). The women had no travel history from any other country, and all of them resided in a hotspot/cluster/containment zone. Most common comorbidities were recorded as hypertension 133 (33.58%), anemia 121 (30.55%), hypothyroidism 99 (25%), diabetes nine (2.27%), and HIV three (2.27%).

**Table 2:** Demographic profile of pregnant women with COVID-19 infection admitted to hospital at the time of delivery.

Characteristics	All patients (n=396)	Percentage (%)
The age group of the patient (years)		
≤19	10	2.52
20-25	169	42.67
26-30	122	30.80
31-35	89	22.49
>35	06	1.51
Education		
No education/pre-school	45	11.36
Primary	103	26.01
Higher secondary	146	36.89
Higher graduation	102	25.75
Occupation		
Housewife	390	98.48
In Job	06	1.51
Gestational age at delivery		
>37 weeks	101	25.50
34-37 weeks	159	40.15
28-34 weeks	122	30.80
<28 weeks	14	3.53
Travel History		
Travel from any other country	None	0.00
Residing in a hotspot/cluster/containment zone/ amongst evacuees from these areas	396	100
Comorbidities		
Hypertension	133	33.58
Anemia	121	30.55
Hypothyroidism	99	25
Diabetes	9	2.27
Chronic lung disease (COPD/Asthma)	None	0.00
Cardiac disease	None	0.00
Renal disease	None	0.00
HIV	3	0.75
Bleeding disorder	None	0.00
Allergy	None	0.00
Addiction		
Alcohol	None	0.00
Smoking	None	0.00

Maternal outcomes

Data on COVID-19 and maternal outcomes are presented showed in Table 3. According to our findings, 187 (47.22%) women were asymptomatic positive for SARS-CoV-2 without any symptoms during their stay in hospital. Out of 396 women, a total of 209 (52.77%) were symptomatic with fever (47.47%), chills (25.25%), running nose (41.66%), congestion (39.89%), cough (34.09%), diarrhea (0.50%), breathing difficulties (5.55%), myalgia (21.96%), vomiting (0.50%), nausea (1.26%), fatigue (45.70%), anosmia (44.19%), sore throat (25.00%), ageusia (28.78%), and anorexia (19.44%) was recorded. All patients were recovered within seven to 14 days and no deaths reported due to COVID-19 pneumonia. The number of vaginal deliveries (n=219; 55.31%) was higher than that of cesarian sections (n=177; 44.69%). A total of 57 (14.39%) experienced preterm deliveries, 15 (3.78%) experienced preterm births with prelabor rupture of membrane (PROM) before 34 weeks, and 11 (2.77%) were reported as preterm births before 37 weeks with PROM. Morphological changes in the placenta were recorded and reported abnormalities in the umbilical cord (n=23; 5.80%), retroplacental clot (n=05; 1.26%), intramural fibrin deposition (n=49; 12.37%); and chronic villi with calcification (n=44; 11.11%). Preeclampsia (n=55; 13.88%), vaginal bleeding (n=17; 4.29%), postpartum hemorrhage (n=10; 2.52%), and pregnancy-induced hypertension (n=6; 1.51%) were also noted as other complications.

**Table 3:** Maternal outcomes of women with COVID-19 infection.

Parameters	All patients (n=396)	Percentage (%)
Asymptomatic	187	47.22
Symptomatic	209	52.77
Fever	188	47.47
Chills	100	25.25
Running nose	165	41.66
Congestion	158	39.89
Cough	135	34.09
Diarrhea	02	0.50
Breath difficulties	22	5.55
Myalgia	87	21.96
Vomiting	02	0.50
Nausea	05	1.26
Fatigue	181	45.70
Anosmia	173	44.19
Sore throat	99	25.00
Ageusia	114	28.78
Anorexia	77	19.44
MODE of delivery		
VD	219	55.31
LSCS	177	44.69
Preterm (n=57)		
Preterm birth before 34 weeks	9	2.27
Preterm birth before 34 weeks with PROM	15	3.78
Preterm birth before 37 weeks	10	2.52
Preterm birth before 37 weeks with PROM	11	2.77
Preterm birth after 37 weeks	12	3.03
Morphological changes in placenta		
Abnormality in umbilical cord	23	5.80
Retroplacental clot	05	1.26
intramural fibrin deposition	49	12.37
Chronic villi with calcification	44	11.11
Hyperplacentosis	None	0.00
Others		
PPH	10	2.52
Hospitalization	186	46.96
PIH	06	1.51
Admitted to ICU	None	0.00
Maternal death	None	0.00
Vaginal bleeding	17	4.29
Blood transfusion received	None	0.00
Need of oxygen	None	0.00
Preeclampsia	55	13.88
Thromboembolic events	None	0.00

Neonatal outcomes

Table 4 showed the neonatal outcomes and complications in the newborns. All the babies were found to be negative for COVID-19 via RT-PCR which indicates that there was no vertical transmission of SARS-CoV-2. A total of 216 (54.82%) babies were male and 178 (45.17%) were females. Newborns (n=202; 51.26%) reported with the normal birth weight as 2.5-3.0 kg and 11 newborn (2.79%) with the range of 3.1-3.5 kg; a total of 155 (39.34%) and 26 (6.59%) newborns were born with low birth weights at 1.6-2.4 kg and ≤1.5 kg. Two (0.50%) intrauterine deaths (IUD) were documented at the third trimester. Additionally, fetal distress (22.33%), meconium-stained liquor (14.46%), post-natal infection with erythematous rashes over face and trunk (0.50%), and diarrhea (0.25%) were recorded as other complications. A total of 22 (5.58%) babies were admitted to NICU due to fetal distress. None of newborns developed fever just after delivery. APGAR score at five min was found to be the standard range for most of the newborns, but low APGAR scores (4-6) at 1 min were observed in 81 (20.54%) babies out of 394.

**Table 4:** Neonatal outcomes from COVID-19-infected mothers.

Parameters	All cases (n=394)	Percentage (%)
Male	216	54.82
Female	178	45.17
Weight of fetus (kg)		
≤1.5	26	6.59
1.6-2.4	155	39.34
2.5-3.0	202	51.26
3.1-3.5	11	2.79
IUD	02	0.50
Fetal distress	88	22.33
Shifted to NICU	22	5.58
Congenital anomaly	None	0.00
Meconium-stained liquor	57	14.46
Post-natal infection	02 (Erythematous rashes over face and trunk)	0.50
Fever	None	0.00
Diarrhea	01	0.25
APGAR score at 1 min		
0-3	0	0.00
4-6	81	20.54
7-10	313	79.44
Total live births	394	99.49

## Discussion

Pneumonia is the most ubiquitous non-obstetric contagious medical condition that can occur during pregnancy. Compared to other bacterial etiologies, viral pneumonia has greater rates of maternal and neonatal morbidity and mortality. Respiratory illnesses, such as H1N1, HIV, SARS, and MERS have been previously documented to cause complications for pregnant women and their fetuses [9,10]. Our study focused on maternal SARS-CoV-2 infections during the third trimester of pregnancy in regard to maternal and fetal outcomes and the vertical transmission of the disease. No evidence of vertical transmission of SARS-CoV-2 was reported in our research findings, as all the other biological samples of mothers such as amniotic fluid, breast milk, cord blood, and placental tissues collected during the pregnancies were tested negative for the presence of SARS-CoV-2. Similarly, there is previous evidence reported that vertical transmission of COVID-19 from mother to child is relatively rare [3,5,8,11,12], although transmission may occur during pregnancy, postpartum, childbirth, and close contact with infected mothers [12]. In a report, no SARS-CoV-2 virus was detected in the breastmilk of 110 pregnant women during the infection, indicating and discovered that breast milk poses no danger of viral transmission via breastmilk into the newborn [11,13]. There is some controversy exists regarding the presence of SARS-CoV-2 in the amniotic fluid; we reported negativity of amniotic fluid from 396 mothers, and several other studies are also reported in support of our findings. The reason for having negative amniotic fluid is due to less stability of the RNA in amniotic fluid than the DNA. RNA is less stable than a deoxyribose molecule because of the presence of the -OH group on the second carbon chain of the ribose sugar. A phosphodiester bond can be broken by a nucleophilic attack because of the -OH group [13-15]. Our study showed that pregnant women were more likely to have a fever, cough, running nose, and congestion, and less likely to have complained of diarrhea, vomiting, nausea, and breathing difficulties, Gao et al. and Makvanti et al. [16,17] also came to similar conclusions in their research. The majority of pregnant females underwent cesarean sections due to fetal distress and reported preterm birth, Wang et al., Makvanti et al., and Gao et al. [15-17] also reported preterm birth as well as cesarean sections due to fetal distress during the SAR-CoV-2 infections in pregnant women. Bobei et al. [18] reported that the rate of cesarean delivery was 82% in their study group as compared to 6% in the control group as well as they also observed and reported a strong correlation between COVID-19 infection and premature birth. Similarly, Liu et al. [19] observed the adverse outcomes, such as premature birth, C-section, and pre-eclampsia occurring more often in COVID-19 patients as compared to the non-infected patients. Placental morphological changes intramural fibrin deposition, chronic villi with calcification, and abnormalities in the umbilical cord were recorded in COVID-19 patients, Sherwani et al. [8] observed similar adverse outcomes in the placenta of pregnant women infected with SARS-CoV-2; however, such outcomes are also associated with the previous pathogenesis, although, both patients with and without placental syndromes can experience placental calcification and the accumulation of calcium-phosphate minerals in placenta tissue [8,20]. In prior reports [8,14-16,20], comparable effects, including postpartum hemorrhage, vaginal bleeding, hospitalization, preeclampsia, and pregnancy-induced hypertension, were also identified during COVID-19 infections, which further demonstrates similarities to our observations. In our study, we recorded neonatal complications such as low birth weight, fetal distress, NICU admission, meconium-stained liquor, and IUD. These facts are very similar to the data reported by Siddiqui et al. [21] who suggested fetal deaths occurred due to respiratory distress as well as the need for NICU to the babies delivered from the COVID-19-infected women. No congenital anomalies were recorded. Calvert et al. stated that there were no or limited congenital anomalies due to COVID-19 infection during pregnancy in a population-based national cohort study [22]. Two IUD (0.50%) occurred during our study which might be, possibly due to the change in fetal heart rate, low APGAR, or fetal distress although until now, there has been a lack of data available regarding IUD due to COVID-19 complications. Kato et al. reported an IUD in a 22-year-old healthy Japanese woman who acquired SARS-CoV-2 infection during her second trimester and lost her fetus in utero due to placental insufficiency brought on by COVID-19 placentitis [12,23]. Some researchers have suggested that fetal death inside the womb is one of the gravest problems that can arise in pregnant women who have COVID-19. While the causes of this occurrence are not clear, however pathological changes to the placenta, particularly a reduction in blood flow to placental tissue and fetal viral infection, may be taken into consideration [12,24]. In our findings, 20.54% of newborns had poor APGAR scores ranging from 4 to 6 at 1 minute. Chao et al. and Yang et al. also conveyed similar findings in their long-term cohort research in which 10,550 neonates born to women infected with COVID-19 during pregnancy had low APGAR scores. In a group of neonates whose mothers had been diagnosed with COVID-19, the risk of having an APGAR score below 7 was 25.4 times higher than the risk for a group of neonates whose mothers had not been diagnosed with COVID-19, indicating that the mother's COVID-19 status affects the neonatal APGAR score [23-26]. Many researchers have claimed that newborns with low APGAR scores may be affected by their mothers' acute symptoms, such as asphyxiation, which can lead to cesarean delivery. Maternal SARS-CoV-2 infection is one of the reasons for placental damage and newborns with lower APGAR scores [24,25,27,28].

## Conclusions

There is no concrete proof of vertical transmission of SARS-CoV-2. Women who had COVID-19 infection during their pregnancy are in serious concern, which can lead to pregnancy complications like preterm birth, PROM, low birth weight, and low APGAR scores. While we were quite interested in recent studies on SARS-CoV-2 vertical transmission, they failed to provide any solid proof of this virus's capacity for vertical transmission from mother to infant. Cases of neonatal outcomes, such as fetal distress, low APGAR score, low birth weight, NICU admission, premature delivery, PROM, and IUD, were recorded in our findings. The results of these studies demonstrate the importance of continuing to track the outcomes of postpartum women and their newborns during these viral infections. To develop an authentic report, a large, prospective, universal study is indeed required to evaluate the long-term outcomes and relationships between COVID-19 in pregnancy and maternal and newborn health. This article is an introduction to determining pregnancy and neonatal outcomes during COVID-19 infections as this research study is still going on at an institutional level. One major limitation of the study is in examining long-term outcomes as patients are lost to follow-up and this may cause a gap in research.
